# Investigating the Impact of a Musical Intervention on Preschool Children’s Executive Function

**DOI:** 10.3389/fpsyg.2018.02389

**Published:** 2018-12-20

**Authors:** Alice Bowmer, Kathryn Mason, Julian Knight, Graham Welch

**Affiliations:** ^1^UCL Institute of Education, University College London, London, United Kingdom; ^2^Creative Futures, London, United Kingdom

**Keywords:** executive function, music, preschool, intervention, assessment

## Abstract

The impact of music interventions on the cognitive skills of young children has become the focus of a growing number of research studies in recent years. This study investigated the effect of weekly musicianship training on the executive function abilities of 3-to-4-year-old children at a London, United Kingdom preschool, using a two-phase experimental design. In Phase 1, 14 children (Group A) took part in eight weekly musicianship classes, provided by a specialist music teacher, while 25 children (Groups B and C combined) engaged in nursery free play. Results of this Phase showed Group A to have improved on two measures relating to planning and inhibition skills. During Phase 2, Group A continued with music classes, while Group B began music classes for the first time and Group C took part in an art intervention. Repeated measures ANOVA found no significant difference in performance improvement between the three participant groups during phase 2; however, the performance difference between groups was nearing significance for the peg tapping task (*p* = 0.06). The findings from this study contribute to current debates about the potential cognitive benefit of musical interventions, including important issues regarding intervention duration, experimental design, target age groups, executive function testing, and task novelty.

## Introduction

### Executive Function

Executive function (EF) is a multidimensional cognitive construct that refers to gaining strategic control over your own mental processes. This could be through inhibiting certain thoughts or actions, or by developing an awareness of your thoughts, feelings, and behavior. EF is closely linked with the construct of metacognition, which is the capacity to know about your own information-processing, monitor your own cognitive performance and to know about the demands made by different kinds of cognitive tasks. It is generally assumed that as children gain metaknowledge about their mental processes, their strategic control will improve (Goswami, 2015). However, an assimilation of the elements that go together to make up EF and a clear understanding of how they link with the processes of metacognition, remain topics of debate.

The elements of EF which are commonly considered to work together to produce cognitive control include working memory, inhibition, and cognitive flexibility (Miyake and Friedman, 2012). During the first few years of life, children develop several broad abilities to hold and manipulate information in the mind, using their working memory. At about 9 months, they have an understanding that objects continue to exist even when they cannot be observed and, shortly after this, children develop the ability to plan and execute two-step tasks. Then, around age 3, they can carry out simple sorting tasks that require them to remember two rules during one activity (Piaget, 1977).

Inhibitory control is a particularly difficult skill for young children to master, requiring them to hold focus on a task, consider the information given and suppress their dominant response before acting. These skills become observable in children from 6 months of age when, for example, they are able to stop themselves from touching something when asked not to by a parent. At age 3, children may still have difficulty with tasks that require inhibitory control of their attention and motor responses; however, important mile-stones are often reached around the ages of 4 and 5 (Carlson, 2005).

Cognitive flexibility refers to our ability to change and adapt our thinking as required by different situations. For example, at around 9 months of age, babies try alternative methods to obtain a toy successfully when the method that they usually use no longer works (Piaget, 1952). Flexibility continues to develop throughout the early years, when children learn different rules for different situations, including the ability to adapt their behavior in a variety of social settings, e.g., being quiet in the library, but noisy in the playground. Generally, children become increasingly accomplished at switching their focus, and adapt to changing rules as they develop.

Executive function development is thought to relate to maturational change in areas of the frontal lobe (Johnson et al., 2009), particularly the prefrontal cortex (PFC) structures which are implicated in a variety of complex behaviors and exhibit considerable growth during early childhood (Gogtay et al., 2004). In general terms, the entire PFC of the brain is dedicated to the memory, planning, and execution of actions. The orbital and medial PFC play a major role in emotional behavior and the control of basic drives, while the lateral PFC supports the temporal integration of information for the attainment of behavioral goals (Fuster, 2001). Both the frontal and prefrontal lobe volumes consistently increase during childhood and adolescence, with the PFC being one of the last regions of the brain to reach full structural development (Kanemura et al., 2003).

While examining associations between developmental changes in the brain and children’s behavior, Bell et al. (2007) found that a shift from global to localized brain activity was evident when making complex responses to EF tasks as children matured. Further evidence from brain imaging studies (see Best and Miller, 2010) confirm significant correlations between structural developments in the brain and improved EF, but the direction and cause of effect remain unclear (Goswami, 2015).

### Problem Behavior and Language Development

Executive function development is associated with language, theory of mind, and non-verbal abilities (e.g., Hughes, 1998), with a range of studies reporting strong correlations between EF and language development (e.g., Gooch et al., 2016). This has led to the hypothesis that children’s use of language may facilitate their performance on EF tasks, and this is consistent with reports of EF deficits found in children with language impairment (see Gooch et al., 2016).

A variety of studies utilizing physiological measures (e.g., heart rate, looking measures, and event-related potentials) have charted significant developmental change in children’s sustained and selective attention throughout infancy (Reynolds and Romano, 2016). For children with attentional disorders, who tend to be impulsive and disruptive in class, inhibitory control is often a key difficulty (e.g., Holmes et al., 2014). This is also the case for children with anti-social behavior disorders which can be accompanied by poor language skills, making the child less effective at controlling their thoughts, emotions and actions via inner speech (Goswami, 2015). Although it can be difficult to distinguish between different types of problematic behavior when children are undergoing such rapid development (Gardner and Shaw, 2008), some children regularly exhibit physical aggression and many find it difficult to form positive interpersonal relationships with peers and teachers (e.g., O’Shaughnessy et al., 2003; Tremblay et al., 2004).

Most children learn to inhibit these problematic behaviors as they develop, but it has been suggested that failure to do so may be an indication of developmental disorders, linked to underdeveloped self-regulation skills (Blair et al., 2005; Nozadi et al., 2015). Although not usually a straightforward process, there is evidence that systematic support for the development of EF skills during preschool and early school years can impact positively on subsequent learning (Diamond, 2012; Blair and Raver, 2014; Goswami, 2015; Jacob and Parkinson, 2015).

### Socioeconomic Status (SES)

Socioeconomic status (SES) is a robust predictor of cognitive performance (e.g., Bradley and Corwyn, 2002). Evidence demonstrates that the development of the neural systems important for language, memory, and cognition are all impacted by a child’s social and economic surroundings (Noble et al., 2005, 2012; Raizada et al., 2008; Hackman et al., 2010; Raizada and Kishiyama, 2010) including those implicated in attention (Neville et al., 2013), memory (Herrmann and Guadagno, 1997; Leonard et al., 2015), and inhibition (Sarsour et al., 2011; Spielberg et al., 2015).

Children who grow up in poverty tend to live in environments that offer less support and stability (Evans, 2004), with fewer opportunities to develop attentional skills and self-control (Dilworth-Bart et al., 2010), both of which are considered to be critical skills for school readiness (Kochanska et al., 1997; Carlson, 2005; Hughes and Ensor, 2008; Morrison et al., 2010). This is consistent with research findings that children from lower SES and at-risk backgrounds often have poorer EF (Mezzacappa, 2004; Ardila et al., 2005; Noble et al., 2005; Hackman et al., 2015; Ursache and Noble, 2016) compared to their wealthier peers. Indeed, Hughes and Ensor’s (2007) study found that social disadvantage already accounted for significant variance in EF ability by age 2.

Fitzpatrick et al. (2014) examined the extent to which EF accounted for socioeconomic-based disparity in school readiness amongst 3- to 6-year-olds, finding that children’s scores on a series of EF tasks predicted their academic ability (as measured by mathematics, reading and vocabulary assessments), after controlling for fluid intelligence and speed of cognitive processing. In line with this finding, children’s preschool executive control difficulties, along with lower family income from early to middle childhood, were found to be robust predictors of later EF difficulties in children aged 7–9 (Raver et al., 2013).

There is also evidence to suggest that EF ability measured early in a child’s life is a predictor of success later in life (Bailey, 2007; Brown and Landgraf, 2010; Davis et al., 2010). For example, a large longitudinal study carried out in New Zealand found that children with lower self-control (relating to attention and inhibition skills) at ages 3–11 tended to have poorer health, earn less and have a higher tendency to commit crimes 30 years later (Moffitt et al., 2011).

Executive function is considered to be important to just about every aspect of life (Diamond, 2013), and appears to be strongly associated with school readiness (Normandeau and Guay, 1998; Blair, 2002; Blair and Razza, 2007; Morrison et al., 2010; Diamond, 2014; Mulder et al., 2017). Today, there is a strong body of evidence to suggest that EF differences play a role in explaining the reported income-based achievement gap (Reardon, 2011; Fitzpatrick and Pagani, 2012; Fitzpatrick et al., 2014; Lawson and Farah, 2017).

### Training and Interventions

The impact of EF on academic achievement highlights a clear disadvantage for children who experience EF deficits (Mason, 2017). Nevertheless, there is evidence that EF can be trained, and reported findings correspond with changes to brain structure and function (Zelazo and Müller, 2010). Early EF training has subsequently been shown to reduce the achievement gap between more-and less-advantaged children (Blair and Raver, 2014, 2016; Ribner et al., 2017). And children from lower-income families, or those with lower working-memory span or ADHD, generally show the greatest improvement in EF as a result of intervention programs (Diamond and Lee, 2011).

In a comprehensive meta-analysis of EF training programs, Diamond (2016) compared a wide variety of methods used to train EFs, including computerized cognitive training, a range of physical activities (e.g., yoga and martial arts), as well as certain school curricula, such as Montessori-based activities and Tools of the Mind. Diamond (2016) found that key elements were consistently present in successful training programs: EF had to be constantly challenged, activity presentation was of a high-quality, and participants spent a good length of time practicing.

However, it can be difficult to determine why improvements are made from some training studies. For example, the computerized working memory training program, *Cogmed*, has been widely studied for it’s capacity to improve working memory. The program’s success was assumed to be due to the computerized games that it uses. However, more recent research by de Jong (2014) found that a core-mentoring component in the *Cogmed* administrator training seemed to account for observed benefits more than the actual games (Diamond and Ling, 2016).

Subsequently, Diamond (2012, 2014, 2016) has suggested that there are many other activities worth investigating for their potential to improve EFs, particularly those with the ability to engage children’s interests, enhance social and emotional development and to provide students with a sense of belonging and social acceptance (Diamond and Lee, 2011). Music making could be a prime vehicle for such experiences (Welch et al., 2014) because it is a multisensory group activity, which simultaneously engages multiple cognitive skills (Miendlarzewska and Trost, 2014).

### Training Transfer

A major debate in the field of training and intervention studies is whether cognitive skill transfer occurs across different domains, i.e., can cognitive skills in one domain transfer to another domain, or increase overall cognitive ability? While near-transfer effects (transfer to tasks within the same domain) have been observed in various training programs, such as preschool computerized working memory (Thorell et al., 2009; Blakey et al., 2015), far-transfer is notoriously difficult to induce and has only been observed through sports, video-gaming and music after demanding specific multi-skill training (for a review, see Green and Bavelier, 2008). Additionally, the credibility of findings has been called into question through a meta-analysis assessing the effect of chess, music and working memory training, where an inverse relationship was found between the size of effect and quality of study design (Sala and Gobet, 2017a). And in pre-schoolers, there are a number of meta-analyses that have found conflicting evidence for the possibility of improving working memory skills through the use of training interventions (e.g., Melby-Lervåg and Hulme, 2013; Schwaighofer et al., 2015).

However, it remains that mechanisms of learning appear to be shared across domains (Goswami, 2007; Green and Bavelier, 2008; Besson and Schön, 2011; Censor et al., 2012; Asaridou and McQueen, 2013; Frost et al., 2015). It is, therefore, possible that systematic music education could boost context-independent cognitive mechanisms, and consequently improve other non-musical cognitive and academic skills (Sala and Gobet, 2017b). This is reported to be the case particularly in subsets of language and EF (Miendlarzewska and Trost, 2014) with some evidence existing for the positive impact of home-based musical activities during 2–3 years of age on aspects of children’s academic attainment at 4–5 years (Williams et al., 2015).

### Benefits of Music Training

Musical learning depends upon the integration of top-down and bottom-up processes (creating sound in the present while remembering it’s relationship to past experience), and it has been suggested that the development of this integration may underlie the enhanced attention and memory processes observed in the musically trained. This is because music making involves the coordination of body movement and auditory perception. Therefore, through musical practice it is possible to refine the connection between movement and auditory areas (Trainor et al., 2009). Other authors have hypothesized that there are two key underlying processes driving the association between music and EF: (1) the willingness to delay gratification in musical instrument learning (e.g., by practicing and working on errors or difficult passages before playing through a piece) (Sternberg, 2005); and (2) that enhanced auditory processing strengthens the ability to detect and deal with conflict (such as detecting and correcting an out-of-tune note) (Slevc and Okada, 2015).

Over the last few decades, a considerable body of research has accrued on differences found between musicians and non-musicians. Differences have been recorded through cognitive testing, brain imaging and behavioral change observed during musical training (Merrett et al., 2013). However, there are a number of contradictory findings (Bilhartz et al., 1999; Costa-Giomi, 1999; Mehr et al., 2013) and results do not always replicate (Miendlarzewska and Trost, 2014).

As one would expect, musically trained children demonstrate better performance than controls in skills closely associated with music, such as auditory discrimination (Forgeard et al., 2008; Hyde et al., 2009), rhythm perception (Moritz et al., 2013; Matthews et al., 2016), rhythmic entrainment (Clayton, 2012; Goswami, 2012; Power et al., 2012; Bhide et al., 2013; Miendlarzewska and Trost, 2014) and fine-motor skill (Overy et al., 2005; Costa-Giomi, 2006; Schlaug, 2015). There is also evidence for near-transfer effects of musical training to lower-level subsets of language ability, including phoneme discrimination (Lamb and Gregory, 1993), phonological awareness (Anvari et al., 2002; Moreno et al., 2009, 2011; Degé et al., 2011) and speech perception (e.g., Francois and Schön, 2011).

Finally, music training has been reported to have positive associations with cognitive domains that are only indirectly related to music, namely vocabulary (Forgeard et al., 2008; Piro and Ortiz, 2009), verbal memory (Ho et al., 2003; Jakobson et al., 2003; Franklin et al., 2008), visuospatial abilities (Stoesz et al., 2007; Patston and Tippett, 2011), mathematical skills (Bahr and Christensen, 2000; Vaughn, 2000; Haimson et al., 2011), IQ (Schellenberg, 2004, 2006, 2011), and overall academic achievement (Fitzpatrick, 2006; Schellenberg, 2006) (for a review, see Miendlarzewska and Trost, 2014).

Evidence of behavioral differences between musicians (i.e., those with significant musical experience) and non-musicians from measures of cognitive testing are consistent with evidence from neuroscience, where there are reports of differences in volume, morphology, density, connectivity, and functional activity across a range of brain regions and structures (Merrett et al., 2013). For example, greater musical expertise has been associated with increased gray matter density in the left inferior frontal gyrus, which is involved in syntactic processing, EF, and working memory, and the left intraparietal sulcus responsible for visuo-motor coordination. Gray matter density was also significantly increased in brain areas involved in visual pattern recognition and in tonal sensitivity (James et al., 2014).

Young et al. (2014), found that providing a child with a musical instrument predicted academic achievement, regardless of poverty level and other socioeconomic factors, which is consistent with research demonstrating a correlation between instrumental music learning and cognitive performance (Eccles and Barber, 1999; Vaughn and Winner, 2000; Broh, 2002; Schellenberg, 2006; Forgeard et al., 2008; Hanna-Pladdy and MacKay, 2011). Hyde et al. (2009) enhanced such findings by showing that structural brain changes in early childhood correlated significantly with improvements of musically relevant motor and auditory skills after only 15 months of musical training.

Jäncke (2009) puts forward the idea of brain plasticity driven by musical expertise and musical training, drawing together evidence from across the different research design approaches. Findings suggest that there are numerous factors influencing when, where and how neuroplasticity occurs in response to musical training (Merrett et al., 2013; Schlaug, 2015).

### Musical Training and Executive Function

What is referred to as a “music” intervention in previous studies ranges from instrumental training, to group musicianship teaching and even computer-based learning. For example, Hyde et al.’s (2009) study included individual keyboard lessons; Sachs et al. (2017) focused on ensemble and group string training; Bugos and DeMarie (2017) trained general musicianship skills using vocal development and various electronic and acoustic instruments; and Moreno et al. (2011) used a computerized program of music training. Therefore, when discussing the impact of “music training” on EFs, the content of interventions should to be carefully considered, including the extent to which studies are comparable. Music should not be considered as a ‘black box’ in which any musical content and processes are suitable.

Four recent musical training studies have shown promising results with children aged between 4 and 6 years. Bugos and DeMarie (2017) examined the effects of a short-term, preschool music program focused on creativity, bimanual gross motor behavior and vocal development on inhibition ability. They assessed 34 children aged 4 and 5 years who were randomly assigned to musical training or a Lego construction intervention, receiving 45 min of training, twice a week for 6 weeks. The music group demonstrated fewer errors on a visual-motor inhibition task post-training when compared to the Lego group. Between group differences were not observed in either response time or answer accuracy on the second inhibition task (involving visual observation/verbal response). In another study, 5-year-old children were pseudo-randomly assigned to complete either a short-term program of computer-based musical training or group painting lessons. Results indicated that the musical training group improved on measures of verbal intelligence and performance on the inhibition task (go/no-go) after 20 days of training, while no significant changes were observed in the painting group (Moreno et al., 2011). Most recently, Jaschke et al. (2018) investigated the influence of a structured music education program on Primary school children using a block randomized longitudinal design. The two music groups (one with and one without prior music experience) were compared to an active visual arts control, as well as a ‘no arts’ inactive control group. Results indicated that children following structured music lessons performed better on tasks designed to measure verbal IQ, planning and a go/no-go inhibition task (similar to Moreno et al., 2011) when compared to controls during follow-up assessments.

Sachs et al. (2017) investigated the effect of music training on EF in 8- to 9-year olds using fMRI measures alongside several behavioral tasks. Children involved in ongoing music training were compared to one group involved in sports and another involved in neither music nor sports. Despite the absence of behavioral differences in performance on EF tasks, the authors reported that children with 2 years of sports or music training displayed a greater activation in brain regions involved in conflict processing when compared to the control group with no systematic training. The results suggest that systematic extracurricular training, particularly music-based training, is potentially associated with changes in the cognitive control network in the brain. For comprehensive reviews of music and EF studies see Dumont et al. (2017) and Jaschke et al. (2018).

## Research Aims

The current study investigated the impact of short-term music training on pre-school aged children’s EF skills. Baseline EF ability was measured using a set of six age-appropriate tasks and the BRIEF-P teacher rating scale (Diamond and Ling, 2016).

The study consisted of two experimental phases:

• Phase 1 compared children’s performance on six EF tasks before and after early music skills training for 40 min per week (Group A) over 8 weeks, while two inactive control groups of children engaged in nursery free play (Groups B and C).
• Phase 2 compared the difference in performance on the same six EF tasks between three groups of children. Group A consisted of children who took part in music training during Phase 1 and who continued with a further 8 weeks of music training (complexity increased). Group B began 8 weeks of the same music skills training as was provided for Group A during Phase 1. Group C undertook 8 weeks of art classes (see Table 1).

**Table 1:** Study design and testing time points.

		Phase 1 (8 weeks)		Phase 2 (8 weeks)	
Group A	Testing time point 1 (TP1)	Music	Testing time point 2 (TP2)	Music	Testing time point 3 (TP3)
Group B		Nursery		Music	
Group C		Nursery		Art	


There were two aims to the research. Firstly, we wanted to see whether early musical skills training improved EF compared with free play in the nursery (Phase 1). Secondly, we included an art-focused active control in Phase 2 so that any improvement in the music intervention groups could be more confidently attributed to the type of intervention, rather than other factors such as increased contact time with an adult.

The unique design of the study builds on the procedures and findings of other research by incorporating both an active and inactive control condition, and implementing carefully considered and structured intervention curricular which included limited use of language (which is discussed further in section “Music Intervention”).

## Participants

The participating nursery was located in West London and integrated as part of a large inner-city Primary school with a diverse population. This nursery was approached for participation in the study as it is situated within the catchment area for Creative Futures, and already had an established relationship with the last author. Approximately 43.9% of children in the preschool class had English as an additional language (EAL), 9.8% had special educational needs (SEN) and 24.4% were eligible free school meals (an indicator of relative poverty) (see Table 2).

**Table 2:** Demographic information for participants.

	Participants	National United Kingdom average ^∗^
% Free school meals	24.4	14.1
% EAL	43.9	20.6
% SEN	9.8	11


*^∗^Data of the national average for free school meal eligibility, English as an additional language, and special educational needs, taken from Department for Education United Kingdom (2017).*

Forty-five children started the study and were pseudo-randomly assigned into three groups – Groups A, B, and C. Children remained in the same groups throughout both phases of the study.

Between the first and second testing time points, three children left the nursery, and three other children were unable to complete the tasks. Therefore, for Phase 1 of the study, Group A *N* = 14, Groups B and C *N* = 25. Total *N* for Phase 1 = 39 children.

At the start of Phase 2, two more children joined the nursery; one was assigned to Group B and the other to Group C. Group numbers for this phase were Group A *N* = 14, Group B *N* = 15, Group C *N* = 12. Total *N* for Phase 2 = 41 children. (see Tables 3 and 4 for participant numbers in each phase).

**Table 3:** Mean age and gender of participant groups in Phase 1.

Phase 1	Total participants	Male	Female	Average age months	Standard deviation
Group A	14	3	11	46.6	4.07
Groups B and C	25	8	17	45.6	2.91
	39	11	28		


**Table 4:** Mean age and gender for participant groups in Phase 2.

Phase 2	Total participants	Male	Female	Average age months	Standard deviation
Group A	14	3	11	46.6	4.07
Group B	15	5	10	45.4	3.18
Group C	12	3	9	46	2.95
	41	11	30		


An analysis of variance was used to ensure that the three participant groups were balanced according to baseline EF ability, using the results of the Behavior Rating Inventory of Executive Function-Preschool, language ability (assessed using subsets of the British Ability Scales) and age.

### Baseline Executive Function Ability (BRIEF-P) (Isquith et al., 2005)

The Behavior Rating Inventory of Executive Function-Preschool (BRIEF-P) is a standardized rating scale designed to measure the range of EF in preschool-aged children. Teachers were asked to rate each child’s EF within the context of their everyday preschool environment. The teacher was presented with sentences about the child’s behavior, such as “When given two things to do, remembers only the first or last” and is asked to respond by circling N (never), S (sometimes) or O (often).

Data from this rating scale were collected at the start of the study in order to help establish a baseline EF measure for each child. BRIEF-P measures were collected on *N* = 41 children from their class teachers (four children were new to the nursery and, therefore, the teachers were not familiar enough with these children to complete the forms).

### The British Ability Scales (BAS-III) (Elliott et al., 1984)

Children were assessed using subsets of the British Ability Scales. These included a picture-naming task as a measure of expressive vocabulary, and an age-appropriate receptive language task.

Results of a one-way analysis of variance (ANOVA) showed that all participant groups were balanced according to baseline EF, language ability, and age. No significant differences were found between the three participant groups for the BRIEF-P [*F*_(2,40)_ = 0.145, *p* = 0.865]; productive vocabulary [*F*_(2,44)_ = 1.189, *p* = 0.315], receptive language [*F*_(2,44)_ = 0.040, *p* = 0.961); nor age [*F*_(2,44)_ = 0.248, *p* = 0.781).

## Materials and Methods

### Intervention Design

The funding for this study allowed for each intervention phase to be delivered for a maximum of 8 weeks. Each weekly music and art session lasted for approximately 40 min, which is the regular session duration for Creative Futures practitioners, who were delivering the classes.

#### Music Intervention

This study was carried out in conjunction with the London-based charity *Creative Futures*^1^ who specialize in providing high quality music and arts programs, often with a pre-school focus. The music intervention consisted of commonly used musical activities suitable for young children and provided in a group context in a familiar space. Prior to the commencement of the intervention, opportunity was taken to explore which types of pre-school musical activity might be appropriate to supporting particular types of EF development (Table 5). All music intervention sessions in both Phases were led by the same Creative Futures practitioner, who selected activities each week from a core curricular. The melodies used during the intervention remained the same throughout, however, each activity increased in complexity over time.

**Table 5:** Examples of music activities included in the intervention and associated areas of EF.

Example musical activity	Associated area of EF
Pitch copycat: teacher sings two pitches to one child and the child’s task is to copy vocally. After listening to teacher and child, all children are to indicate if the child’s response was the same or different, using a physical (non-verbal) hand gesture of two fists for the same and one fist and an open hand if different.	Working memory, inhibition
Melody recognition: different musical themes representing actions (e.g., galloping on a horse or riding a train) are played on a piano to which children act out different movements. Children are required to switch between actions as the music changes.	Working memory, inhibition, cognitive flexibility
Musical phrasing: In a circle, children walk around to a melody, and stop when the music ends to count eight beats. During the eight-beat count, they must swap places with another child in the circle.	Working memory, inhibition, planning
Musical anticipation: A rectangular mat is placed at the back of the room. Children dance around the room to a four-phrase melody played on a piano. During the last phrase, children have to move themselves close to the mat, ready to jump onto it on the last note of the melody.	Working memory, inhibition, planning


The use of language, for both instruction and singing, was purposefully limited during the intervention. This was to ensure that any effect found might be primarily attributed to musical experience, rather than an increase in language input. However, the young children who took part in the study were not instructed to limit their language use and were free to communicate and respond as they wished.

#### Art (Active Control Condition)

The art classes consisted of weekly age-appropriate 40-min practical sessions based on different techniques themed around particular artists’ work. The art classes were designed and taught by a Creative Futures early years specialist art practitioner, who was also instructed to limit her use of language.

## EF Assessment

Designing tasks that clearly assess one specific aspect of EF (see Garon et al., 2008) is complex, because they often rely upon one another during performance on a given task. For example, many inhibition tasks also rely on using working memory. Therefore, if failure is recorded, it is difficult to determine within which component of EF it occurred. To overcome this problem, studies sometimes adopt an aggregate approach to assessment, using a number of different tasks designed to capture individual elements of EF (Miyake and Friedman, 2012; Devine et al., 2016). However, EF ‘piggy backs’ on other complex cognitive functions, such as language, making it difficult to tease apart EF deficits from other areas of cognition, such as motor, auditory, visual or verbal perception. Deficits can occur in any one of these areas, making it challenging to attribute failure on a task as purely an issue with EF. These problems are especially pertinent in research with pre-schoolers when many of these skills are in the early stages of development.

With this in mind, multiple assessment tools were used in order to provide a more thorough assessment of EF, however, scores from the tasks were not used to produce a composite score. Tasks were chosen that were widely reported to be appropriate measures of EF for this age group. They were also selected for their reasonable administration time and appeal to preschoolers. Children were assessed at all three testing time points using the same six EF tasks. Each child was tested individually, spending 15 min with one researcher (the first author) and 15 min with another (the second author) to avoid fatigue and to help maintain the child’s interest. Instructions for each task were kept simple, and were supported with the use of gesture when required, to ensure children’s understanding. No child refused to participate in any of the tasks, and many children reported enjoying the tasks.

### Peg Tapping (Luria, 1966)

Involving both rule learning and switching, this task was first used with children (Diamond and Taylor, 1996). It requires both the ability to hold two things in mind: (1) the rule to tap once when experimenter taps twice and (2) the rule to tap twice when experimenter taps once, as well as the ability to exercise inhibitory control over one’s natural tendency to mimic what the experimenter does. Using a wooden dowel, the child is asked to tap twice, immediately after the experimenter taps once, and to tap once immediately after the experimenter taps twice. The child is given praise or correction after each practice. There is no limit to the number practice trials offered. When the rules are understood, the experimenter proceeds with 16 test trials in a pseudo-random order. Children are not given feedback during any of the test trials. Scoring for children who completed the task had a possible range of 0–16 points. Children for whom the task was aborted received a score of -1. Common errors included: (1) complied with only one of the two rules; (2) tapped many times regardless of what the experimenter did; and (3) copied the experimenter, rather complying with the rule. This task has adequate test–retest reliability, with a reliability coefficient of 0.80 (standard error = 0.03) (Lipsey et al., 2017).

### Baby Stroop (Hughes and Ensor, 2005)

This task is designed to assess inhibitory control. Children were presented with a normal-sized cup/spoon and a baby-sized cup/spoon. The experimenter randomly assigned each child to either Group A (cup trials followed by spoon trials) or B (spoon trials followed by cup trials). In the control phase, children must name the large cup/spoon “mummy” and the small cup/spoon “baby.” During the second phase, children must use the labels incongruously (Roman et al., 2016). The 12 trials are presented in a pseudo-random order, with scores ranging from 0 to 12.

### Dimensional Change Card Sort (DCCS) (Zelazo, 2006)

The Dimensional Change Card Sort is a standard procedure for assessing cognitive flexibility in early development. Children have to sort cards according to a rule – either color or shape. They are shown cards with boats or rabbits on them, either blue or red in color. Two sorting trays are placed side-by-side. Target cards are fixed to the back of each tray, one showing the image of a red rabbit, and the other a blue boat. The experimenter points and verbally names the two target cards. In the pre-switch phase, children are asked to sort six cards according to their color, after two demonstrations given by the experimenter. Cards were presented to the child in a pseudo-random order. In the post-switch phase, children were asked to sort the cards by shape. A mark from 0 to 6 was given for the pre-switch trials and 0 to 9 for the post-switch phase (1 mark for congruent cards and 2 marks for incongruent cards). Scores ranged between 0 and 15 marks.

### Trucks (Hughes and Ensor, 2005)

This task involves rule learning, working memory and rule switching and was used as a measure of cognitive flexibility. Each child was tested with an eight-trial pre-switch phase. The child is shown a pair of similar trucks on one card. They are then asked to choose one of the two trucks to win them a reward (one raisin per correct answer) and asked to remember that truck to continue to win them the reward in later trials. The child is then shown another card with two new trucks and asked to do the same thing. Six pseudo-random trials follow and the child moves onto the post-switch phase if four of the last five trucks are correctly identified. The post switch phase consists of eight trials with trucks mounted on different colored card. The child is told that in this game they must choose the other truck to win the reward. One mark was awarded per correctly identified truck, with a range of 0–16.

### Tower of London (Shallice, 1982)

This task was used to measure complex planning. A wooden base block with three pegs and three colored blocks was presented to the child alongside an iPad with an image of a block arrangement. Children had to reproduce six different block arrangements by moving only one block at a time and using the minimum number of moves needed. Trials consisted of two, three, and four move problems. Extra moves were allowed and, if the child stopped, the experimenter allowed one re-try from the original starting place. Scores reflect the number of correct trials that each child was able to complete and were marked out of six.

### Spin the Pots (Hughes and Ensor, 2005)

The Spin the Pots task was developed to assess working memory and inhibition in young children. The child was shown eight distinct “pots” which are set up on a Lazy Susan tray, and then invited to help the researcher place attractive stickers in six of the eight pots. The tray was then covered with a cloth and spun. Following this, the cloth was removed and the child choose a pot with the aim of finding all six stickers without error. Each choice was recorded and the child congratulated/encouraged before moving on to the next trial. Fixed spatial cues could not be used due to the rotation of the cups. Children were allowed a maximum of 16 trials and the task ended when all six stickers had been found. The task was scored as 16 minus the number of errors.

## Phase 1 Methods

All 45 children underwent baseline testing (TP1) on the six EF tasks. Assessments were conducted 1:1 in a cordoned off area of the children’s usual nursery setting, by two researchers (the first and second authors); one researcher being blind to which experimental group the children were assigned to. During Phase 1, Group A (music intervention) took part in weekly 40-min musicianship classes for 8 weeks, while Groups B and C continued with their regular nursery playtime. A register was kept for each class to track attendance. After 8 weeks, all children were retested on the six EF tasks (TP2).

Three children left the nursery, and three children were unable to complete the assessments before TP2 testing. Their data were, therefore, excluded from the analysis. Consequently, Phase 1 data consists of Group A, *N* = 14 (music intervention) and Groups B and C, *N* = 25 (control group).

**FIGURE 1:**
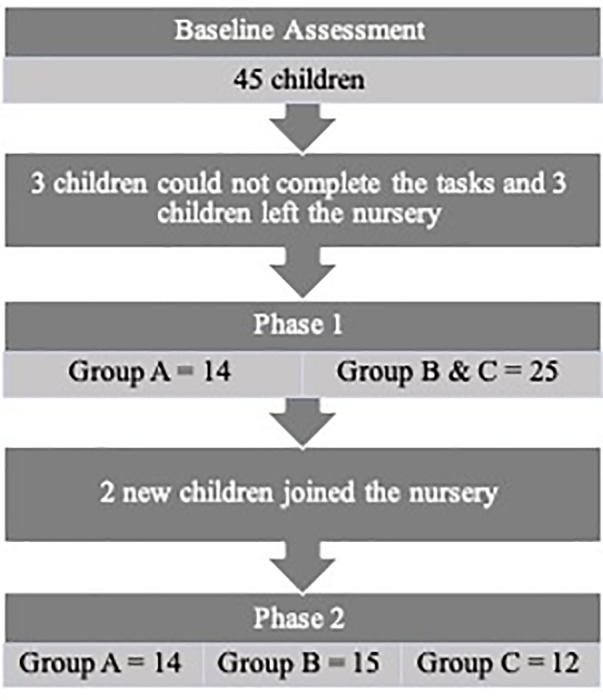
Flow chart of study and participant numbers for phase 1 and phase 2.

If children were unable to do any task, the tester abandoned the task and that child’s data was not included in the analysis of that particular task. Subsequently, there was no missing data to be accounted for (see Figure 1 for information on participant numbers in each phase).

### Results of Phase 1

Data from TP1 (baseline) and TP2 were analyzed using a 2×2 (group × testing time point) repeated measures ANOVA. The mean scores and standard deviations for both groups are shown in Table 6. Results of the repeated measures ANOVA are shown in Table 7.

**Table 6:** Means and standard deviations (in parenthesis) for the intervention and control group on each EF test at TP1 and TP2.

Test	Executive function	Group A TP 1	Group A TP 2	Groups B and C TP1	Groups B and C TP2
Peg tapping ^∗^	Inhibition	6.07 (5.28)	11.07 (5.68)	6.68 (5.71)	7.62 (5.38)
Baby Stroop^∗^	Inhibition	8.77 (3.44)	7.00 (3.83)	7.00 (4.31)	8.88 (3.47)
Card scoring	Cognitive flexibility	9.21 (3.19)	10.00 (3.19)	8.72 (2.69)	10.96 (2.62)
Trucks	Cognitive flexibility	5.79 (4.12)	6.79 (5.32)	6.68 (4.09)	7.08 (4.09)
Tower of London ^∗^	Complex planning	2.71 (1.64)	4.07 (1.14)	3.04 (1.62)	3.56 (1.00)
Spin the pots	Working memory	11.29 (4.05)	12.50 (2.38)	11.32 (2.12)	12.96 (1.72)


*^∗^Indicates tasks where a significant difference in change scores was found between the intervention and control group.*

**Table 7:** 2 × 2 repeated measures ANOVAs.

Source	*df*	MS	*F*	*p*	Effect size
Peg tapping	1,37	159.392	8.379	0.006^†^	0.190
Peg tapping × group	1,37	73.238	3.988	0.053^∗^	0.097
Baby Stroop	1,35	0.047	0.004	0.949	0.000
Baby Stroop × group	1,35	55.993	4.918	0.033^∗^	0.123
Card sorting	1,37	41.080	8.376	0.006^†^	0.185
Card sorting × group	1,37	9.490	1.935	0.173	0.050
Trucks	1,37	8.795	0.511	0.479	0.014
Trucks × group	1,37	1.615	0.094	0.761	0.003
Tower of London	1,37	15.811	20.365	0.001^‡^	0.355
Tower of London × group	1,37	3.145	4.050	0.051^∗^	0.099
Spin the pots	1,37	36.557	7.231	0.011^†^	0.163
Spin the pots × group	1,37	0.813	0.161	0.691	0.004


*MS, mean squares; effect size = $\eta_{p}^{2}$. ^∗^p < 0.05, ^†^p < 0.01, ^‡^p < 0.001.*

Due to issues during testing, it was necessary to exclude the data from two children for the Baby Stroop task. Therefore, for this analysis, intervention group *N* = 13 and control group *N* = 24.

### Phase 1 Discussion

A main effect of test was found for all but two of the assessments, which shows that children improved over time on the majority of the tests. There was no significant main effect for the Baby Stroop or Trucks tasks, indicating no significant improvement in the children’s performance on these assessments between TP1 and TP2. However, for the Baby Stroop task, a significant interaction between task and participant group was found [*F*_(1,35)_ = 4.918, *p* = 0.03, η^2^ = 0.123]. The data in Table 6 shows that for this task, the control group’s performance modestly improved at second testing; however, the intervention group’s performance fell slightly. The researchers experienced issues with the administration of this task, which may explain this somewhat surprising result. The poorer performance of the music group on the Baby Stroop task during TP2 testing may be due to a variety of factors. A couple of the children who had been able to do the task during baseline testing refused the premise of the task during second testing, while others had performed at ceiling throughout testing and, therefore, their improvement was unable to be seen over time. These potential testing issues are addressed further in the discussion.

Significant interactions between group and test were found for the Tower of London task and the peg tapping task, both at *p* < 0.05. For these tasks, (measures of planning and inhibition, respectively) the group of children who had 8 weeks of musical intervention showed improvement on the assessments at a significantly higher rate than the control group. Given the relatively short duration of Phase 1 (8 weeks), these significant findings are indicative of a positive effect of the music intervention, and support findings from other studies suggesting that improvements in EF skills can be found after relatively short intervention times. However, the lack of an active intervention group weakens this conclusion, as the observed differences between groups may be due to other factors, such as increased contact time with enthusiastic and engaging adults. Therefore, the addition of an active control group in Phase 2 aimed to address this issue.

## Phase 2 Methods

Two new children joined the nursery and were included in Phase 2 of the study. Therefore, for Phase 2, *N* = 41 children participated. During Phase 2, the original group (Group A, *N* = 14) continued with music classes for a further 8 weeks. Group B (*N* = 15) began 8 weeks of music classes with the same *Creative Futures* musician as Group A, following a very similar program to that of Group A in Phase 1. Group C (*N* = 12) received 8 weeks of 40-min art classes with a different *Creative Futures* visual arts practitioner and were considered to be the active control group. As with Phase 1, a register was kept for all of the classes to monitor attendance, with all children attending at least half of the sessions.

### Results of Phase 2

Data presented for Phase 2 in Tables 8 and 9 compare EF improvement across the three participant groups between TP2 and TP3, using a 3×2 (group × testing time) repeated measures ANOVA.

**Table 8:** Means and standard deviations (in parenthesis) for the three participant groups on each EF test at TP2 and TP3.

Executive function task	Group A	Group B	Group C
Peg tapping TP 2	11.07 (5.68)	7.40 (5.84)	8.00 (5.59)
Peg tapping TP 3	13.71 (4.39)	12.93 (4.22)	9.83 (4.82)
Baby Stroop TP 2	7.00 (3.83)	8.43 (4.22)	9.73 (2.10)
Baby Stroop TP 3	10.92 (1.61)	10.86 (1.83)	11.00 (1.84)
Card sorting TP 2	10.00 (1.96)	10.67 (2.55)	11.00 (2.70)
Card sorting TP 3	11.71 (2.76)	11.87 (2.85)	11.08 (2.31)
Trucks TP 2	6.79 (5.32)	8.40 (4.01)	5.58 (3.99)
Trucks TP 3	9.57 (4.13)	9.80 (5.12)	7.42 (4.27)
Tower of London TP 2	4.07 (1.14)	3.40 (1.18)	3.58 (0.79)
Tower of London TP 3	4.86 (0.66)	4.20 (1.01)	4.00 (0.95)
Spin the pots TP 2	12.50 (2.38)	12.93 (1.53)	13.00 (1.86)
Spin the pots TP 3	14.36 (1.39)	13.80 (1.66)	13.67 (1.97)


*NB. Three children were unable to complete the Baby Stroop task. Therefore, for this task data presented are Group A (*N* = 13), Group B (*N* = 14), and Group C (*N* = 11).*

**Table 9:** 3 × 2 repeated measures ANOVAs.

Source	*df*	MS	*F*	*p*	Effect size
Peg tapping	1,38	226.237	26.509	0.001^‡^	0.411
Peg tapping × group	2,38	26.395	3.093	0.057	0.140
Baby Stroop	1,35	121.481	14.498	0.001^‡^	0.293
Baby Stroop × group	2,35	10.636	1.269	0.294	0.068
Card sorting	1,38	20.290	8.109	0.007^†^	0.176
Card sorting × group	2,38	4.432	1.771	0.184	0.085
Trucks	1,38	81.807	6.645	0.014^∗^	0.149
Trucks × group	2,38	3.594	0.292	0.748	0.015
Tower of London	1,38	9.054	18.264	0.001^‡^	0.325
Tower of London × group	2,38	0.301	0.607	0.550	0.031
Spin the pots	1,38	25.957	13.501	0.001^‡^	0.262
Spin the pots × group	2,38	2.752	1.431	0.252	0.070


*MS, mean squares, effect size = $\eta_{p}^{2}$. ^∗^*p* < 0.05, ^†^*p* < 0.01, ^‡^*p* < 0.001.*

### Phase 2 Discussion

**FIGURE 2:**
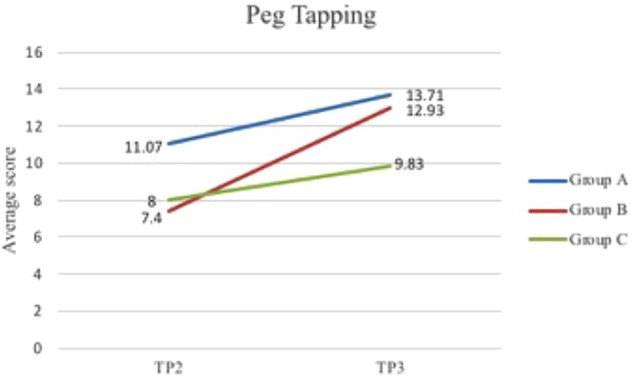
Mean scores for all participant groups on the peg tapping task at TP2 and TP3.

The ANOVAs showed a significant main effect for all tasks, indicating that the children’s performance improved significantly on all tasks between TP2 and TP3. There was no significant interaction between participant group and task for any of the EF assessments during Phase 2. However, the interaction between the peg tapping task and participant group was approaching significance at *p* = 0.06. Mean scores from the three participant groups show that Group B, (who began music classes in this phase), show the greatest improvement in peg tapping ability post-musicianship training. This is shown in Figure 2 below. It is also notable that all groups had similar baseline scores for peg tapping at TP1 (6.07 for Group A, and 6.68 for Groups B and C), and by the end of Phase 2, Groups A and B have significantly higher scores than Group C – the only group not to undergo musicianship training.

## Discussion

### Overview

The study investigated any potential far-transfer effects of a short-term music intervention on pre-school aged children’s EF skills. In Phase 1, the group of children who received eight weekly music classes showed greater improvement on the Tower of London task (a measure of complex EF and planning) and peg tapping (a measure of inhibitory skill) than their peers who remained in regular nursery playtime. However, these differences in performance improvement were small, with small effect sizes and were not maintained during Phase 2, when an active control condition was introduced. Nevertheless, there was a trend for greater improvement in the two music intervention groups on the peg tapping task during Phase 2, which suggests that the music sessions may have had some impact on children’s inhibitory motor response skills. These findings are consistent with previous studies which have found small or inconclusive effects of music training on children’s cognitive skills (e.g., Miendlarzewska and Trost, 2014).

Explanation for this finding may lie in the content of the music intervention classes. The activities were uniquely designed to use as little spoken language as possible. Consequently, it was necessary for the children to pay close attention to the music teacher while following her gestures and instructions, which required active sustained attention throughout every music session. While there was no intervention activity which was directly comparable to the peg tapping task, the children would often have to infer the rules of a particular ‘game’ by watching the teacher and learning what their response should be. Similarly, the peg tapping task made comparable cognitive demands on the children – learning a rule, identifying and remembering the correct response, and inhibiting an incorrect prepotent response. Additionally, the researchers found that the peg tapping task was one of the easier tasks to undertake with children who had relatively low language ability, due to the minimal language required to explain the task’s rules. It may be that the groups who attended the music classes had better performance on this task post-intervention due to repetitive practice of musical activities requiring attention, motor, and inhibitory skill.

Previous studies of far-transfer effects of music interventions have so far yielded mixed or inconclusive results (for a review see Dumont et al., 2017). The findings from this study contribute to current debates about the potential cognitive benefit of musical interventions, including important methodological issues such as intervention duration, experimental design, EF testing (including tools) and task novelty.

### Intervention Duration, Content, and Quality

Several studies have found a positive effect of music interventions on EF after a relatively short period of time [e.g., Moreno et al. (2011), daily for 8 weeks; Mason (2017) twice a week for 5 weeks; Bugos and DeMarie (2017) twice a week for 6 weeks]. However, there is currently no consensus on the quantity of intervention needed to reliably produce changes in children’s EF, although it is assumed that longer duration and frequency will produce exponentially more robust effects (Diamond and Lee, 2011). Intervention group size is also an important consideration for training effectiveness. Previous studies have ranged from whole class interventions (e.g., Bodrova and Leong, 1996) to small, differentiated groups (e.g., Mason, 2017), or individual instruction (e.g., Moreno et al., 2011). The group sizes in the current study were moderate, with a maximum of 15 children, to ensure engagement with the activities. The interventions were also conducted over a comparatively short period of time, with only one session of music per week. Despite this, positive effects were found for children’s planning and inhibitory skills on two tasks, with moderate effect sizes.

In the current study, musical training was delivered through group activities, which focused on pre-school appropriate games, allowing the children to develop and build on their skills each week. Due to the strong association between language and EF, the intervention involved minimal spoken language with the intention of reducing any potential compounding effects of language on EF change. This intervention feature was unique as music classes are usually taught through the use of language. Additionally, program content, quality and delivery are of fundamental importance. The content of the intervention reported here was carefully designed by experienced, early years music practitioners and delivered by a highly trained music teacher. Therefore, the authors are confident of the consistency and quality of the music sessions.

### Strengths and Limitations of the Study

A strength of the study was the inclusion of two phases: one with an inactive control and the other with an active control condition. Phase 1 results revealed significant post-intervention change in planning and inhibitory skills for the music intervention group compared to the controls. However, the significant findings from Phase 1 were not replicated in Phase 2 when an active control condition was included, despite there being a trend for improved inhibitory skill. This study is unique in that it consisted of two phases, with the second phase acting as an immediate semi-replication of the first. Without the replication of findings from Phase 1, Phase 2 demonstrates the importance of careful experimental design, and the inclusion of active control conditions to reduce the risk of false-positive results.

Another strength of this study was the use of a variety of assessments, avoiding reliance on a single task to measure a particular element of EF, which is especially important when attempting to assess EF in young children. This issue is highlighted by Zelazo and Müller (2010) who suggest that simple EF tasks designed to probe one specific aspect of EF may, in fact, tap into multiple component processes (see Lehto et al., 2003).

An additional strength was the focus on preschool aged children. Some authors have called for an increase in studies of EF development for this age group and younger (e.g., Wass, 2015), as it is in the early years of children’s development that brain plasticity is at its most malleable, and higher-order cognitive skills are rapidly developing. It is also an age phase where the development of effective EF skills is essential for school readiness, and identification of children with deficits in EF development is vital in order to provide them with effective support and intervention. However, there are some methodological limitations that arise when working with this age group, including there being a limited number of assessments with wide enough sensitivity to track changes in EF ability over short time periods.

Limitations to the study include a short duration and relatively infrequent delivery of the intervention, and the number of participants. Specifically, the research was restricted by the number of children available to participate at the nursery; group sizes which were restricted by the number of children who could be comfortably taught at any one time; and the necessity of having three different groups in the research design. This resulted in modest participant numbers in each group, and therefore a lack of statistical power which is likely to have impacted the findings. However, the intervention duration was comparable with that of previous studies (as addressed in section “Phase 1 Discussion”). Future research plans would ideally include a larger sample size, with a longer intervention duration.

#### Task Novelty

Generally, children did not refuse to play any of the games during all three test time-points. However, there were issues with the novelty of the tasks. During testing, it was observed that the Baby Stroop task in particular had some re-testing issues. The results of this task during Phase 1 revealed a lowering of performance for the music group. Observation of the raw data showed that three children performed at ceiling on this task during baseline testing, and so no improvement could be seen, while other children who could do the task at baseline, subsequently refused to accept the rule during second testing. We believe the task was not novel enough during the second and third testing time points in order to sustain children’s attention, particularly as the stimuli remained the same. It was the only task where performance fell, suggesting an issue with test/re-testing, i.e., the task itself was perhaps not appropriate for re-testing within this relatively short timeframe. This is not unique to the current study and has been previously addressed as an important issue in EF testing when using a repeated measures intervention design (Hughes and Graham, 2002; Müller et al., 2012). Additionally, the children may have been performing at chance on the task, as they struggled to understand its premise, and are already susceptible to making scale errors at this age.

Developing tasks for young children that are simultaneously feasible for some to achieve, while remaining challenging for others, is one of main testing issues in the pre-school age group. Lack of a ‘fine-grained’/nuanced scoring system for some tasks only allows children to pass or fail a task, without providing any insight into their developmental progress. This was demonstrated in the current study, where group variability meant that some children were unable to complete the tasks and had to be removed from the study, while others performed at or near ceiling. Additionally, the children were a diverse, heterogeneous group, and while this was representative of many inner-city areas, the high percentage of children with special needs (SEND) and for whom English was an additional language (EAL) likely also had an impact on testing. For example, during the baseline picture vocabulary testing, some of the children switched between responding in English and their home native language, which is common for bilingual children in this age group (Hoff et al., 2012).

### Future Directions

Familiarity with tests is an issue for any short-term intervention study. In future related studies, it is advisable to maintain the novelty of the tasks by using alternative stimuli during retesting, while maintaining the same task procedures. In particular, the use of simple, concise and consistent instructions for tasks is important, especially when working with children whose first language may not be English. Additionally, consideration of fatigue effects from multiple tests of EF and testing at particular times of the day is needed, especially with pre-school children. Task retesting windows are a known problem in intervention studies, particularly when they are of a short length (*c.f.* see discussion by Chan et al., 2008). Limited information is available on retest windows for EF tasks. However, the current study demonstrated that the majority could be represented at 3-month intervals without complaint from the children.

Although there was not enough scope in the current study, it would be beneficial for future studies to continue to investigate the impact of music interventions, both with and without language, in order to examine the relationship between music, language and EF. Other studies have found individual music and language interventions to have comparable effects (Bhide et al., 2013; Cohrdes et al., 2018), strongly indicating overlap between the two domains.

As children’s EF is developing rapidly during the preschool years, the challenge for future studies will be to tease apart the impact of the intervention from regular EF development. One of the ways that this could be achieved is with the inclusion of both active and inactive control conditions, as was included in the design of the current study. The quality of intervention delivery can also impact on the outcome, particularly if provided by a motivated and skilled practitioner. Careful consideration should, therefore, be given to recruiting the most appropriate person to deliver the intervention and, ideally, they should be blind to the aims of the study.

## Conclusion

Results from both Phases of this study show promising indications of the potential impact of musical intervention on pre-schoolers EF skills, particularly in inhibitory control. It is possible that exposure to intervention on a more regular basis, with a larger sample size, and for a longer amount of time, would produce more robust results. This study broke new ground in that it demonstrated the importance of including an active control in EF intervention research with young children. Future studies may also benefit from careful consideration of intervention design and how particular features of intervention activities map on to the specific elements of EF being measured. This will help to stimulate and strengthen discussion about what specific elements of music contribute to the enhancement of EF skills.

## Data Availability Statement

All children participated following written consent from their parents/carers, in line with standard research protocols of the British Educational Research Association (BERA) and the ethics policy of the charity Creative Futures.

## Ethics Statement

This study was carried out in accordance with the recommendations of “Creative Futures, United Kingdom” with written informed consent from all subjects. All subjects gave written informed consent in accordance with the Declaration of Helsinki. The protocol was approved by “Creative Futures, United Kingdom: www.creativefuturesuk.com/policy”.

## Author Contributions

AB, KM, GW, and JK have equally contributed to the conception and design of the work. Data collection as well as analysis and interpretation have been undertaken by AB and KM under supervision of GW.

## Conflict of Interest Statement

The authors declare that the research was conducted in the absence of any commercial or financial relationships that could be construed as a potential conflict of interest.

## Footnotes

1 https://www.creativefuturesuk.com
